# Development of an Interactive, Web-Delivered System to Increase Provider–Patient Engagement in Smoking Cessation

**DOI:** 10.2196/jmir.1721

**Published:** 2011-10-18

**Authors:** Rajani S Sadasivam, Kathryn Delaughter, Katie Crenshaw, Heather J Sobko, Jessica H Williams, Heather L Coley, Midge N Ray, Daniel E Ford, Jeroan J Allison, Thomas K Houston

**Affiliations:** ^1^Division of Health Informatics and Implementation ScienceQuantitative Health SciencesUniversity of Massachusetts Medical SchoolWorcester, MAUnited States; ^2^VA eHealth Quality Enhancement Research InitiativeBedford VAMCBedford, MAUnited States; ^3^Division of Continuing Medical EducationSchool of MedicineUniversity of Alabama at BirminghamBirmingham, ALUnited States; ^4^Division of Gerontology, Geriatrics and Palliative CareDepartment of MedicineUniversity of Alabama at BirminghamBirmingham, ALUnited States; ^5^Division of Clinical Immunology and RheumatologySchool of MedicineUniversity of Alabama at BirminghamBirmingham, ALUnited States; ^6^Division of General Internal MedicineSchool of MedicineUniversity of Alabama at BirminghamBirmingham, ALUnited States; ^7^Department of Health Services AdministrationSchool of Health ProfessionsUniversity of Alabama at BirminghamBirmingham, ALUnited States; ^8^Division of General Internal Medicine, Johns Hopkins School of MedicineBaltimore, MDUnited States; ^9^Center for Health Quality, Outcomes & Economic Research (CHQOER)Bedford VAMCBedford, MAUnited States

**Keywords:** Smoking cessation, general practice, family practice, public health informatics, user interfaces, randomized controlled trial, health services research, web-based services

## Abstract

**Background:**

Patient self-management interventions for smoking cessation are effective but underused. Health care providers do not routinely refer smokers to these interventions.

**Objective:**

The objective of our study was to uncover barriers and facilitators to the use of an e-referral system that will be evaluated in a community-based randomized trial. The e-referral system will allow providers to refer smokers to an online smoking intervention during routine clinical care.

**Methods:**

We devised a four-step development and pilot testing process: (1) system conceptualization using Delphi to identify key functionalities that would overcome barriers in provider referrals for smoking cessation, (2) Web system programming using agile software development and best programming practices with usability refinement using think-aloud testing, (3) implementation planning using the nominal group technique for the effective integration of the system into the workflow of practices, and (4) pilot testing to identify practice recruitment and system-use barriers in real-world settings.

**Results:**

Our Delphi process (step 1) conceptualized three key e-referral functions: (1) Refer Your Smokers, allowing providers to e-refer patients at the point of care by entering their emails directly into the system, (2) practice reports, providing feedback regarding referrals and impact of smoking-cessation counseling, and (3) secure messaging, facilitating provider–patient communication. Usability testing (step 2) suggested the system was easy to use, but implementation planning (step 3) suggested several important approaches to encourage use (eg, proactive email cues to encourage practices to participate). Pilot testing (step 4) in 5 practices had limited success, with only 2 patients referred; we uncovered important recruitment and system-use barriers (eg, lack of study champion, training, and motivation, registration difficulties, and forgetting to refer).

**Conclusions:**

Implementing a system to be used in a clinical setting is complex, as several issues can affect system use. In our ongoing large randomized trial, preliminary analysis with the first 50 practices using the system for 3 months demonstrated that our rigorous preimplementation evaluation helped us successfully identify and overcome these barriers before the main trial.

**Trial:**

Clinicaltrials.gov NCT00797628; http://clinicaltrials.gov/ct2/show/NCT00797628 (Archived by WebCite at http://www.webcitation.org/61feCfjCy)

## Introduction

Smoking is the number 1 behavioral health problem and preventable cause of death in the United States [1-5]. Among its innumerable morbidities, smoking is responsible for approximately one-third of all cancer deaths [6]. Patient self-management interventions that can easily be disseminated, such as self-help materials, computer-tailored printouts, interactive voice-response systems, quitlines, and, more recently, smoking-cessation websites [4,7-14] can potentially engage much greater numbers of smokers [15]. Unfortunately, these interventions are underused [16]. For example, as few as 3.5% of adult smokers access quitlines per year [17]. These patient self-management interventions are often deployed as public health interventions and are not well connected to clinical medicine.

Because the majority of smokers (70%) see a provider at least once per year [18], point-of-care referrals could greatly increase use of publicly available self-management smoking-cessation interventions. A recent study using proactive fax referrals to quitlines demonstrated an increased number of patients using these services [19]. Although clinical providers report limited time and competing demands as barriers to referring patients to smoking-cessation resources, they also acknowledge the role of a single source of referral, additional support, referral coordinators, and reimbursement for tobacco counseling in aiding the intervention process [20]. A system seamlessly linking the physicians, nurses, and patients within a clinical microsystem may be more effective in reducing barriers to physician referrals. Further, increasing standard protocols, data collection, and feedback between individuals in the microsystem can maximize patient-centered care [21-23].

This paper describes the preimplementation evaluation of a provider e-referral system (ReferASmoker.org). ReferASmoker.org will be used in a nationwide randomized trial that will recruit 160 primary care physician practices and test the e-referral functions [24]. A system intended to be used in a clinical setting must overcome the barriers that may impede its success. These barriers may be software usability issues or problems integrating with the standard processes of care. Our “how-to” report demonstrates how small, rigorously conducted, multistep preimplementation evaluation can positively affect the success of the larger study. Our preliminary analysis in the main trial shows that our evaluation approach successfully identified many barriers in the study’s formative stages, and we were able to overcome them before the main study trial.

## Methods

ReferASmoker.org is a point-of-care e-referral portal that allows providers to e-refer smoking patients to an online smoking-cessation portal. The ReferASmoker.org system (http://www.ReferASmoker.org) can be accessed using the email address reviewer@nih.grant and the password “review”.

### Study Design

**Figure 1:**
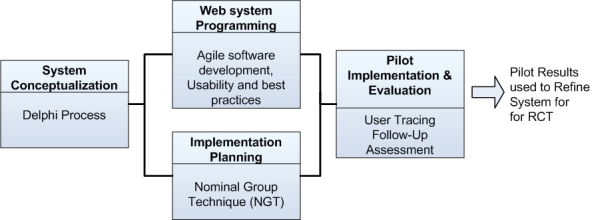
Development stages of ReferASmoker.org (RCT = randomized controlled trial).

Our four-step usability and pilot testing approach consisted of (1) system conceptualization using the Delphi technique to identify key functionalities that would overcome barriers in provider referrals for smoking cessation, (2) Web system programming and refinement using agile methodology and think-aloud usability testing, (3) implementation planning using the nominal group technique (NGT) for the effective deployment of the system in practices, and (4) pilot testing to identify practice recruitment and system-use barriers (Figure 1).

### Setting and Sample

For system conceptualization, we recruited experts (clinical, informatics, and tobacco control) from multiple academic institutions. For usability and pilot testing, we selected practices that would represent the sample in our planned randomized trial. Thus, physicians and nurses from community-based practices across several states in the United States were recruited. For our implementation planning sessions, we recruited physicians from a university setting. Our study was approved by the institutional review boards at the University of Alabama at Birmingham, the Johns Hopkins University School of Medicine, and the University of Massachusetts Medical School.

### Phase 1: System Conceptualization

To conceptualize the system, we used a modified Delphi process [25,26], a systematic forecasting method for reaching consensus regarding prediction of usability and feasibility. It is a useful communication strategy that provides a structured process for the reliable and creative exploration of ideas suitable for decision making. Controlled opinion feedback sessions are used to establish expert consensus without certain social interactive behaviors that can hinder opinion forming in a typical group discussion [25]. 

A panel of 8 experts that included physicians and psychologists with expertise in health services, tobacco control, and informatics participated in the Delphi process. Our goals were to identify the major limitations of current smoking-cessation systems, along with identifying areas to refine in order to maximize physician engagement in the referral of patients to smoking-cessation resources within our system. We conducted three face-to-face discussions over a period of 3 weeks, and in-between email discussions augmented the process. One investigator (TKH) was responsible for synthesizing a literature review and presenting to the panel in the first face-to-face meeting. The same investigator was responsible for summarizing meeting minutes, distributing them by email, and then organizing the email discussions for the next round of the face-to-face discussions in the Delphi process.

### Phase 2: Programming and Usability testing

#### Agile Software Development

Agile software development was used to iteratively strategize and plan the programming of the ReferASmoker.org e-referral system. Unlike the traditional approach of specifying system requirements fully at the outset of development and then undertaking programming, the system is developed in units after an overall strategy is formulated. In each agile phase, a short-term goal is set for developing a unit of the system, followed by team development of the unit, including requirements, design, programming, and testing. Agile software development is advantageous because developers can adapt to changing requirements based on the short-term goal setting and collaboration. This approach has also been demonstrated to reduce development time and risk [27].

#### Web System Programming

The ReferASmoker.org Web-based system was programmed using Microsoft’s ASP.NET version 3.5 (Microsoft Corporation, Redmond, WA, USA) and C# technology. Microsoft SQL Server version 2000 was used as the database. We used programming best practices in the form of design patterns and modular architecture. Design patterns have been used over the years to solve software development problems. Originally introduced by the Gang of Four [28], these design patterns have evolved, and many are being used in developing Web systems.

Frameworks make it easier to use patterns. Specifically, we used the Web Client Software Factory (WCSF) version February 2008 [29], which is a .Net-based framework introduced by Microsoft. In the WCSF, the Web user interface is programmed using the model-view-presenter (MVP) design pattern [30]. The MVP pattern splits the Web interface into three layers: (1) a *model* that defines the data to be displayed or acted upon in the user interface, (2) a *view* that displays the model and routes user commands (events) to the presenter, and (3) a *presenter* that acts upon the model and the view such as formatting the data for display in the view. The modular approach of MVP makes it easier to modify the Web layer without affecting other areas of the system and to unit test the system for programming errors. In addition to the use of MVP in the Web layer, WCSF divides the rest of the system into business modules and foundational modules. Business modules guide the programming of the business logic of the system. The foundational modules are used to program the data access and reusable functions of the system. The modular approach of WCSF makes it easier to make programming changes to the system, as each layer is only loosely connected to the others. This approach also makes it easier to independently test each layer for programming errors using mock data.

To implement data access, we used the combination of NHibernate and Castle ActiveRecord frameworks (version Release Candidate 1) [31,32]. These frameworks guide consistent and structured data access from the database using object-relational mapping (ORM). ORM is a technique that maps the relational data structure of the database into an object-oriented structure [33]. Castle ActiveRecord leverages NHibernate functions and implements the active-record pattern [34,35], a database-related design pattern in which a database table is modeled in terms of a class and a row of the database table is modeled by an instance of the class. The properties of the class correspond to the columns of the table. The ORM and the active-record pattern provide a consistent model and make it easier to access and manipulate the database from within the programming language. Another advantage of this approach is that programming time can be reduced by reusing many of the Castle ActiveRecord and NHibernate methods such as FindAll (find all records) or FindByProperty (find records related to a property such as all activities of a patient) to query for data without having to write Structured Query Language (SQL) queries.

#### Usability Testing

Usability of the system was assessed using the “think-aloud” approach [36-38]. In this approach, while participants are reviewing the system’s content and interacting with the program, they are asked to vocalize thoughts, feelings, and opinions. The think-aloud approach gives an insight into how the user approaches the interface and what considerations the user keeps in mind when using the interface. 

Think-aloud interviews were conducted with community providers (physicians and nurses, n = 3). A semistructured interview was used to collect input, and optional prompts were used if a provider did not continue to vocalize during the usability interview. The interview was conducted over the phone by study staff trained in the think-aloud protocols. Each interview was recorded and transcribed. Providers were asked to sign onto the ReferASmoker.org system, go through the registration process, and navigate through the site while making comments about their perceptions of the visual layout, as well as the location of options and functions within the system.

### Phase 3: Implementation Planning

Once the primary processes were identified, we conducted an NGT session to collect feedback on the referral system and plan for implementation in practices. NGT is a highly structured, multistep, consensus-building procedure often used in formative research to elicit and prioritize group responses to a specific question. It is a consumer-oriented formal brainstorming or idea-generating technique used to foster creativity and to effectively prompt group members to articulate meaningful disclosures [39,40]. 

The study was conducted with a panel of experts (n = 9) that included health services researchers, and internal medicine and family practice providers. Using case scenarios, we introduced the goals of the study to the panel, as well as the proposed key components of the Web-based system identified in the process-mapping Delphi. The NGT sessions followed a standard protocol of solicitation of comments, discussion, and ranking of comments by level of importance. Questions posed were as follows: (1) What can we do to help you integrate ReferASmoker into your work clinic?, and (2) What would help you remember to use ReferASmoker?

### Phase 4: Pilot Implementation and Evaluation

We tested implementation of the system to identify recruitment barriers and areas of refinement in the system. We recruited providers from family practice clinics to participate in the pilot study. Practices in the pilot were representative of participants in our planned larger trial. Using methods from a previously published randomized trial [41], we mailed 400 interest surveys that included a brief letter of introduction and a 1-page survey to determine a provider’s interest and eligibility to participate in the project. Providers could respond to the interest survey online, by fax, or by mail using a prepaid, self-addressed envelope. If chosen for inclusion, providers were mailed a practice survey with a $150 incentive for completion. 

Once the practice survey was completed, participants were mailed instructions on how to access and register on the website. We then measured the participant’s usage of the system by tracking their interactions with the website. These data included the pages visited as well as the number of patient referrals on the system. After a period of use, each enrolled practice was contacted by telephone for follow-up; we assessed potential barriers and facilitators to future implementation at that time.

## Results

### Phase 1: System Conceptualization

We presented results of the literature review to our multidisciplinary research panel with expertise in health services, tobacco control, and informatics. Through the Delphi, our panel identified three key functionalities that would serve to overcome gaps in smoking-cessation referrals in clinical practices. 

First, the research panel identified the importance of passive referrals such as information prescriptions in cessation efforts [42]. The panel recommended that providers use an information prescription approach with the ability to refer patients directly into an electronic system at the point of care. This Refer Your Smokers functionality would require a patient identifier, such as an email address, to be entered into a secure Web form or desktop client. Then, the system would automatically send active email reminders to patients encouraging participation.

Second, sustained cessation is difficult, and providers do not always have the benefit of observing the positive impact of increased counseling and referral activities. Their attention to smoking cessation has little short-term positive reinforcement. In other referral processes for preventive care, there is often a *p* *roximal* *o* *utcome*—a report of the result of screening. These reports (eg, results of a Pap test) produce a feedback loop and allow for an observable impact. Thus, our panel recommended creating practice reports that detail (1) the number of patients referred, and (2) the number of referred patients actually participating. These rates could be compared with other participating providers and potentially increase referrals.

Third, although many clinic-based interventions refer patients to public health services, such as quitlines, we noted almost no literature on referrals from public health interventions back into clinical care. Recent advances in prescription pharmacotherapy to aid smoking cessation make referral back to the provider for pharmacotherapy even more important. Thus, public health interventions should include content emphasizing the importance of seeking clinical treatment when a patient is ready to quit. The patient website should provide information about how to talk to your doctor about quitting and information about medications. For facilitating linkage back to clinical services, the panel recommended that patient and provider be connected via a secure messaging system. Thus, patients would be supported in the follow-up process, and providers could more easily assist with treatment and arrange follow-up.

In summary, based on the findings of the Delphi process, we conceptualized the following: (1) the system should support direct referral at point of care, (2) the system should provide continuous reports on patient activities to encourage continued participation of the providers, and (3) the system should support linkage of patients back to clinical services.

Additional functionalities were conceptualized to support the core functionalities noted above, including (1) a “quick-start” guide to train providers to use the system, (2) educational cases and materials to enhance provider knowledge about smoking cessation, (3) downloadable tools to support practice workflow (eg, posters to be used as cues for referral), and (4) methods for engaging providers longitudinally in the system (eg, a “headlines” section with evolving content, continuing education credit for educational cases, and an email reminder system to encourage referrals). 

### Phase 2: Programming and Usability testing

#### Website Functions

**Figure 2:**
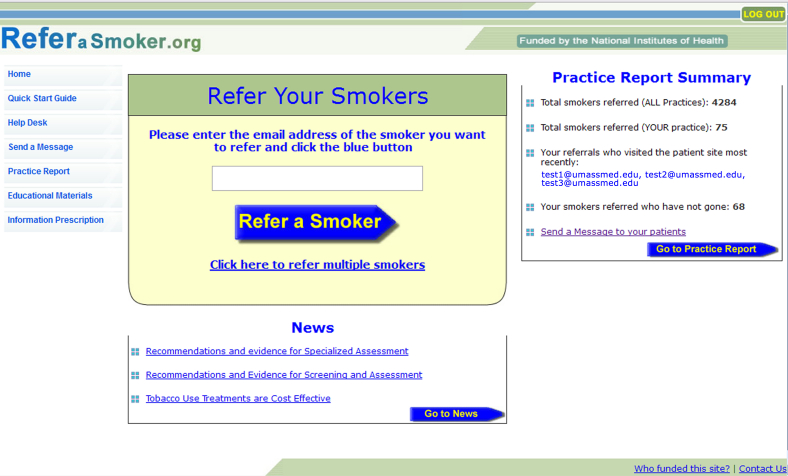
ReferASmoker.org Web-based system home page.

The ReferASmoker.org Web-based system was programmed using ASP.Net and C# technology (Figure 2). The following functions were developed: Refer Your Smokers, practice reports, secure messaging, and registration.

The core Refer Your Smokers function allows providers to proactively refer and enroll patients in the smoking-cessation system during the clinical encounter. To refer a patient, the provider logs into the ReferASmoker.org system and enters a willing patient’s email address. Patients can be referred in multiples or one at a time. The patient referral triggers several automated processes: (1) the patient’s email is entered into the database of the patient online smoking-cessation system, enabling the patient to register and login to the patient system, (2) the system links the patient with the appropriate practice and provider, enabling the practice reports and secure messaging functions, and (3) a series of automated emails is sent to encourage the patient to login to the smoking-cessation system.

**Figure 3:**
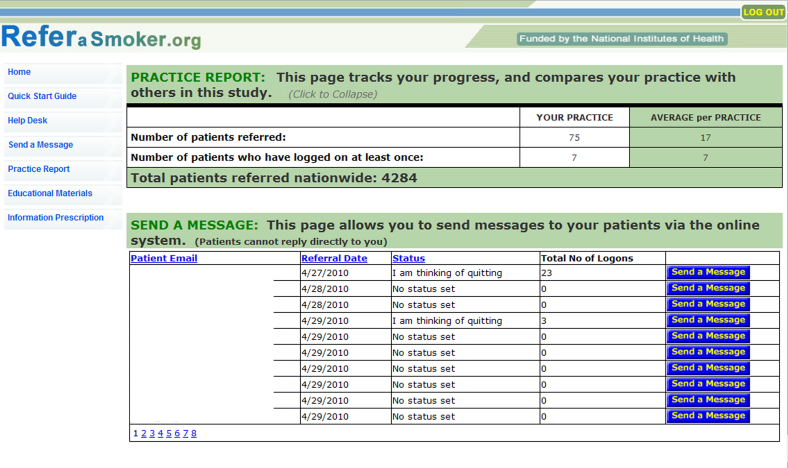
Sample ReferASmoker.org Web-based system practice report.

The practice reports feature was specifically designed to increase observability of provider impact in supporting patients who smoke to take steps to improve their health by quitting (Figure 3). This function allows providers to monitor their patient smoking-cessation activities in real time. Several components of activity for providers are detailed, including (1) the numbers of patients referred, (2) the number of referred patients actually participating in the program, and (3) a comparison of these rates with other participating providers from practices across the country.

The secure messaging function was designed to enhance provider–patient communication. Providers can send messages to their patients to encourage use of the patient portal in their smoking-cessation efforts. For convenience, the system provides message templates, but providers have the option to customize them during their registration into the ReferASmoker.org system. A link to the secure messaging function is located within the ReferASmoker.org system so that providers have enhanced communication capabilities with their patients, who also receive this benefit on the portal. Providers can also initiate message threads within the secure messaging system.

In addition, we developed a toolbox of educational materials, interactive cases, and news headlines on the website. These materials were developed to supply providers with more general resources and materials to aid in the implementation of smoking-cessation strategies. The interactive cases were followed by questions testing comprehension of the information. Links were embedded in the interactive feedback to redirect the provider to different sections of the education materials to obtain additional information. On completion, providers earned 1 American Medical Association Physician’s Recognition Award category 1 continuing medical education credit for each case. A registration process was created for a provider to register to the system using an email and password combination. The registration process included online consent, a survey, and two customizable email messages to the patient. Once the registration was completed, the provider could login to the system on the home page using the email password combination.

#### Web System Programming

Guided by the WCSF, we programmed the system using a modular and flexible architecture. We further divided the modules of the WCSF into the data access layer that enabled structured database access, the service layer that provided a collection of reusable functions, and the business process layer that orchestrated the functionality of system.

In the data access layer, using Castle ActiveRecord and NHibernate tools, we created ORM mappings between database tables in the SQL database to C# classes. The ORM mappings also included the relationships that exist between tables in the SQL databases. For example, both a table for the list of providers and another table for the list of practices were developed. A many-to-one relationship exists between these tables (ie, a provider can belong to many practices and a practice can have many providers). The relationship was replicated in the ORM mappings using the BelongsTo and HasMany attributes of Castle ActiveRecord. The provider class possessed a property indicating that the provider BelongsTo the practice class and the practice class had a property indicating it HasMany providers.

In the services layer, we programmed “reusable” data query functions and common utilities that are used throughout the application. The reusable data query functions leverage the data access layer to perform query functions such as select, insert, and update. For example, the system contained provider functions that perform such operations as select all providers belonging to a practice, find the practice of the provider, or find the randomization of a particular practice. The utility functions included methods to send emails and encrypt and decrypt data. A SendEmails function was used throughout the system to send emails to patients, including transmission of secure emails when a provider uses the secure messaging function on the website or transmission of automated emails to encourage registration from the system. The provider and patient identifiers were stored in encrypted form in the database. For this, algorithms to encrypt and decrypt the provider and patients identifiers appropriately were programmed in the DataEncryption function.

In the business process layer, we programmed the business logic of the system—that is, a series of tasks that orchestrated the services to realize the functionality of the ReferASmoker.org processes, such as Refer Your Smokers, provider feedback, and secure messaging. For example, the Refer Your Smokers process performed several tasks that need to occur when a provider refers a patient, including (1) determining whether the patient was already referred in the database, (2) if a patient was already in the database, informing the provider that the patient was already referred, and (3) if it was a new patient, adding the patient’s information (email, referring provider and referring practice information, referral date, and emails assigned for transmission from the provider to the patient) and informing the provider that the referral process was successful.

#### Usability Testing

Feedback acquired through the think-aloud usability testing was categorized into three themes: (1) registration and login process, (2) general layout, and (3) specific features. As the providers went through the registration process, several issues were identified. First, the instructions indicating that a new user must register and choose a password before using the system were not altogether clear. Second, the providers expressed displeasure with the system automatically assessing the strength of the password provided. Third, instructions for completing the registration survey and particular questions within the instrument were not clearly understood. Finally, the length of the registration process prevented completion of the usability testing process and was seen as a potential barrier to use of the system in practice.

Regarding the overall layout of the system, the providers indicated that the website was user-friendly and the various components self-explanatory. Providers expressed particular interest in the news headlines and education components of the system. Providers commented positively on the simplicity and ease of the Refer Your Smokers function. Providers were also pleased that once a referral was made, the system automatically emailed patients to remind them to visit the site. However, concern was expressed regarding the usefulness of the system for patients without email. 

The practice reports were also believed to be of great utility. Providers remarked that the nationwide comparison of referrals and the real-time activity of their patient panel could serve as motivation to improve. The suggestions for improvement included adding an attention-getting visual to draw the eye to the status column, listing the most active patients at the top of the status report, and adding a mechanism that would announce a patient’s first visit to the website or when a particular patient was doing very well or very poorly.

Providers were enthusiastic about the secure messaging function’s potential to engage patients in their own care but provided several thoughts. First, the providers indicated value in the ability to print, download to an electronic medical record, or otherwise archive the messages sent for the medical records. Documenting these communications without additional work was seen as very important for proper follow-up and for possible reuse or modification in the future. Next, providers appreciated both the opportunity to use a preestablished message template that tailored content based on where the patient is in the quit process and the ability to make the messages more personal. Finally, providers commented that it might be beneficial for patients to have the ability to respond to the provider messages to engage them more in their care and in their quit processes, but they also acknowledged that a two-way communication path within the system could prove burdensome for many providers. 

With regard to the educational toolbox, providers suggested that the various products should be labeled separately for convenience of location on the website. No matter how useful the information, busy clinicians would not spend precious time searching for the information. Further, more information for providers was suggested, including a quick-facts sheet with the latest statistics about smoking and links to the most relevant and recent evidence. Print options for all materials, including other treatment information (for patients), were also suggested.

 Based on the feedback from the usability results, we made several changes to the system. An easily visible button with the text “NEW USERS! Please click this button to register” was created on the home page to clarify new-user registration instructions. The instructions on the survey page were also clarified. We removed the password strength feature on the username and password creation page. To reduce the additional step of logging in the system after registration, users were redirected to the home page after completing registration. In response to the comments on the practice reports, we created a practice report summary on the home page that contained the following information: (1) a count of the numbers of smokers referred using the system by all practices and the number of smokers referred by the current practice, (2) emails of the last 3 patients of the current practice who were active on the patient site, and (3) the number of smokers of the current practice who have not visited the patient website. We did not create two-way secure messaging between the provider and patient because we felt that this will add additional burden to the providers. To improve the educational materials section, we further classified the materials into three sections: practice forms, interactive cases, and patient education. The first two sections grouped materials for increasing the knowledge and awareness of the provider. The latter sections, though delivered to the provider, contained materials for the provider to use for educating patients. 

### Phase 3: Implementation Planning

From the NGT session, we identified that several cues to action would be needed to implement ReferASmoker.org in practices, including workflow items and continuous reminders. First, NGT participants emphasized the importance of communicating with the practice through a contact person. This person would serve as the liaison with the practice over a set period of time to inquire about patient recruitment or any other questions or concerns with the system. Second, incentives for participation (e-referrals) were recommended. Third, a continuous communication plan, including both mail and email campaigns, was suggested for ongoing practice engagement. Participants indicated that regular emails would update participants about study progress and provide other information relevant to smoking cessation. Emails with embedded weblinks would provide convenient access back to the system. Fourth, in addressing practice workflow issues, it was recommended that hardcopy materials be sent to the practice to facilitate collection of patient email addresses and website instructions. Finally, NGT participants suggested that successes be appropriately celebrated, perhaps with emails of congratulations and gratitude to practices that logged into the study. 

### Phase 4: Pilot Implementation and Evaluation

In the pilot implementation, 25 practices out of 400 responded to a mailed survey indicating that they were interested in the project, and all of them were mailed a consent form. Of the 25 practices, 8 returned the consent form and were then mailed a practice survey. Of these 8 practices, 7 returned the survey and were given access to the ReferASmoker.org system. Out of those, 6 providers from 5 practices registered with the system, and 5 of them logged into the system. Initially, no providers referred patients. The principal investigator of the study contacted each of the practices by phone to encourage them to use the website. After the call, 1 provider used the referral function to refer 2 patients. Among these, 1 patient visited the patient website. 

Telephone calls from the principal investigator to enrolled practices were not included in the original pilot implementation and evaluation protocol. However, it became important to elicit information from providers at this stage that could prove helpful in the main trial. We attempted phone contact with all 6 enrolled providers and succeeded in talking with 4. The providers reported barriers and facilitators to practice implementation. Overall, the providers liked the system and thought the intervention was a good idea, but had trouble implementing the system. The staff in the practices constantly changed and newer staff members were unaware of the study. Practices also did not remember whether they had registered with the system. Practices also forgot to e-refer because of lack of visual cues to the intervention. One provider summarized this succinctly, stating “I guess it’s out of sight, out of mind.” Providers encouraged cues to action, with suggestions for a waiting or examination room display that would serve as reminders to refer or to activate patients to talk to them about smoking cessation. Providers were not sure whether the system would be applicable to all patients. All providers agreed that an implementation budget would provide incentives for use of the system.

## Discussion

In the preimplementation stage of a nationwide study of an interactive, Web-delivered system to increase provider and patient engagement in smoking cessation, we conducted a rigorous planning and evaluation of the system. The primary purpose of our preimplementation evaluation was to identify the strengths that might be used to promote the program, and weaknesses that might be mitigated prior to initiating the main study. We conceptualized and developed e-referral functions in Web-based form. We report the functions we developed and the results of our usability testing in the Results section. We evaluated the Web system and the implementation plan rigorously with community-based providers. Our approach involved four phases: (1) system conceptualization, (2) agile programming and think-aloud usability testing, (3) implementation planning (using the NGT), and (4) lessons learned from pilot implementation in 7 physician practices. Table 1 summarizes the identified barriers and facilitators to practice implementation based on our evaluation work. In the section below, we focus on the implementation protocol changes that will be used in the main trial to address the four primary barriers we uncovered in the pilot testing.

**Table 1:** Identified issues related to e-referral system implementation

Issue	Barrier	Facilitator	Identified by	Identification stage
Difficulty contacting the practice and lack of study champion	X		Study team	Pilot
Lack of training	X		Study users (providers)	Pilot
Registration difficulties	X		Study team and study users (providers)	Think-aloud usability
Lack of motivation and start-up incentives	X		Study team and study users (providers)	Think-aloud usability and pilot
Forgetting to refer	X		Study users	Pilot
Ease of system use		X	Study users (providers)	Think-aloud usability and pilot
Perceived potential to affect care		X	Study users (providers)	Think-aloud usability and pilot

The first barrier was the difficulty contacting the practice and lack of study champion. With no champion identified at each practice, we were constantly speaking with or leaving messages for different staff members, who had little sense of ownership of or urgency in the process. This breakdown in communication was made more complicated with staff turnover, a reality in most medical offices. In order to overcome this particular barrier, we modified the study protocol to include a request for each practice to identify two staff members to serve as implementation coordinators. These implementation coordinators will be the primary contacts for the practice and will work closely with our study personnel. Their responsibility will be to implement and promote the study intervention in the practice. Two implementation coordinators will allow for backup in the event that one individual is unavailable or leaves the practice. Our study personnel will communicate with these implementation coordinators to confirm practice information, hold training sessions, answer any questions, and provide feedback. 

Second, we identified that successful implementation required training and assistance with registration in the system. Pilot practices reported that the system was easy to use, but with no one trained at the practice to complete the registration process and refer patients and to champion others through the process, the task went undone. Consequently, we increased study personnel and created a proactive helpdesk to provide training and help with registration. In the main study, our staff will initiate contact with each practice within 2 weeks of receiving the returned consent form. Study personnel will verify practice information and schedule a training and registration call for each of the implementation coordinators. During this call, our staff will walk the implementation coordinators through the actual registration process. The study personnel will be on hand to answer any questions. Following registration, study personnel will review the process for referring patients, getting the implementation coordinators to enter a dummy referral to have the full experience of the ease of referral. Each implementation coordinator will be encouraged and provided information to train others in the office to also register and refer patients. Following the training call, study personnel have planned a booster call to verify receipt of printed materials sent and answer any questions that may have arisen in the first few attempts to refer. If no referrals have been made yet, our staff will assess any reasons for no referrals and encourage implementation coordinators to use the system. 

 The third barrier was a lack of motivation and start-up incentives. It became abundantly clear that motivation to participate was low. We focused on increasing both extrinsic and intrinsic motivation. Pilot data immediately indicated that financial incentives would spur participation. Additional funds from the American Recovery and Reinvestment Act has enabled us to provide honoraria to participating practices and individual staff members who complete training and surveys.

Finally, we learned that clinicians within participating practices simply forgot about the study and the need to refer patients through the system. We believe that with convenient reminders we will be able to activate them to use the system. In addition to calling the implementation coordinators to aid them in the registration process and answer any questions, we will increase the work-flow support. We also improved the printed information prescription pads sent to practices for distribution to their patients simultaneously with their online referral. The “Information Rx” that was used in the pilot was small, about the size of a regular prescription pad, and simply provided an optional patient handout. The new and improved pad is spiral bound and has easy check-off boxes with duplicate pages. The bottom half of the first page will be given to the patient. The top half will be returned to study personnel, and the duplicate copy will be ready to place in a patient medical record file. Further, the information prescriptions for the intervention arm have a space for the providers to write the patient’s email address. Additionally, posters to serve as visual stimulation to use the system, posters to encourage patients to talk with their provider about quitting, and 1-page instruction sheets outlining the steps for referring patients will be sent to participating practices.

To increase intrinsic motivation and to maximize the brief phone contact with practices, study personnel will incorporate a concept called motivational interviewing into each interaction. Miller and Rollnick define motivational interviewing as a client-centered, directive method for enhancing intrinsic motivation to change by exploring and resolving ambivalence [43]. Key concepts involved in motivational interviewing are accurate empathy, reflective listening, and overcoming ambivalence, with the ultimate goal of facilitating some changed behavior. From our pilot study, we learned that implementation coordinators must face an increased workload because of their participation in the study enrollment, training, and implementation, especially in system registration and patient referral. Using motivational interviewing techniques, our staff will be better able to communicate effectively with implementation coordinators by identifying and overcoming their ambivalence. As an example, if a study staff member has attempted to contact a particular implementation coordinator on multiple times and failed, it may be a natural inclination to reflect negatively on that statement, which can be highly detrimental to the relationship with that particular office. Focusing on a positive reflective statement might increase the likelihood of the practice either becoming or continuing to be a happy participant in the study and increase positive feedback, which will, in turn, facilitate study task completion. 

### Results of Implementation Protocol Changes

Because of our implementation protocol changes, we were successful in engaging practices and improving participation in the nationwide trial. We measured rates of referral and patient participation in the first 3 months of practice engagement. To date, we have analyzed data from the first 50 e-referral practices. Practices’ mean e-referral rate was 14 (SD 13.63). In the first 3 months, the maximum number of referrals by a practice was 62, and 3 practices did not refer. Per practice, the patients mean registration rate was 3.4 (SD 5.09). The maximum number of patients registered with a practice was 28, and 13 practices did not yet have any patients registering.

### Strengths and Limitations

In preparation for a nationwide randomization trial testing an e-referral process for referring patients to a smoking-cessation system by providers, we detail the rigorous steps taken to develop the Web-based e-referral system. At each step of the development process, we applied user input to conceptualize and refine the system. Although the numbers of users are low, the multiple and comprehensive nature of the interactions and data collected provided significant information on which to improve the system’s usability. The results of the mini pilot study gave us critical insight into the recruitment and use barriers that our randomized trial must overcome to succeed.

### Conclusion

Our how-to report demonstrates how a small, rigorously conducted, multistep preimplementation evaluation can affect the success of a larger study. To gain valuable information regarding potential improvements to an interactive, Web-delivered provider–patient system to increase engagement in smoking cessation, we used a multidimensional approach to conceptualize, develop, implement, and test the product and process. The results of this rigorous process led us to make significant changes to the practice implementation approach study, prior to its nationwide randomized, controlled trial. After refining our information system with usability testing, we further uncovered serious barriers to implementation: lack of study champions within the practice, lack of training and assistance in use of the system, and lack of motivation to participate. We identified several improvements to address and made changes to the main study protocol before trial implementation. Our preliminary analysis with the first 50 practices using the system for 3 months demonstrates the preimplementation evaluation was successful in overcoming the barriers to recruiting and retain study participants. 

## Abbreviations

MVP: model-view-presenter
NGT: nominal group technique
ORM: object-relational mapping
SQL: Structured Query Language
WCSF: Web Client Software Factory
